# Modelling and estimation of genotype by environment interactions for production traits in French dairy cattle

**DOI:** 10.1186/1297-9686-44-35

**Published:** 2012-11-26

**Authors:** Bérénice Huquet, Hélène Leclerc, Vincent Ducrocq

**Affiliations:** 1 INRA, UMR1313 Génétique Animale et Biologie Intégrative, F-78352 Jouy-en-Josas, France; 2 Institut de l’Elevage, 149 rue de Bercy, 75012 Paris, France

## Abstract

**Background:**

Genotype by environment interactions are currently ignored in national genetic evaluations of dairy cattle. However, this is often questioned, especially when environment or herd management is wide-ranging. The aim of this study was to assess genotype by environment interactions for production traits (milk, protein, fat yields and fat and protein contents) in French dairy cattle using an original approach to characterize the environments.

**Methods:**

Genetic parameters of production traits were estimated for three breeds (Holstein, Normande and Montbéliarde) using multiple-trait and reaction norm models. Variables derived from Herd Test Day profiles obtained after a test day model evaluation were used to define herd environment.

**Results:**

Multiple-trait and reaction norm models gave similar results. Genetic correlations were very close to unity for all traits, except between some extreme environments. However, a relatively wide range of heritabilities by trait and breed was found across environments. This was more the case for milk, protein and fat yields than for protein and fat contents.

**Conclusions:**

No real reranking of animals was observed across environments. However, a significant scale effect exists: the more intensive the herd management for milk yield, the larger the heritability.

## Background

Two main opportunities are available to improve production traits in dairy cattle: through the modification of herd management and/or the genetic level. Except when it is necessary to choose a local breed for a specific environment (such as the Abondance breed in the French Alps), these two opportunities are generally considered separately, as in genetic evaluation. Indeed, they imply the absence of genotype by environment (G*E) interactions, i.e., the breeding value of an animal is assumed to be the same regardless of the environment in which it will be raised. Dealing with this situation, some breeders question the efficiency of current breeding schemes for their own particular management system. Thus, the objective of this study was then to estimate G*E interactions for production traits (milk, protein, fat yields and fat and protein contents) in French dairy cattle. The overall objective was to assess whether these interactions could be an opportunity to better adapt animals to their environment. G*E interaction studies raise three main questions: How to define the genotype? How to describe the environment? Which model to choose in order to estimate G*E interactions? This study used an innovative description of herd environment: Herd Test Day (HTD) profiles, which are by-products of a test day model evaluation. Two models, a multiple-trait and a reaction norm model were tested.

## Methods

The approach consisted of two steps. The first step dealt with the definition of herd environment through HTD profiles. This was done across breeds (Holstein, Normande and Montbéliarde) rather than within breed because two herds with different breeds could share the same type of environment. The second step was a G*E interaction analysis. As genetic evaluations are within breed, G*E parameters were estimated within breed.

### Description of the environment: Herd Test Day profiles

The methodology used to describe herd environment from HTD profiles was described in [1]. The main difference with this previous study is that we worked here with a larger dataset. A short description of the main steps involved and results obtained follows.

**Figure 1:**
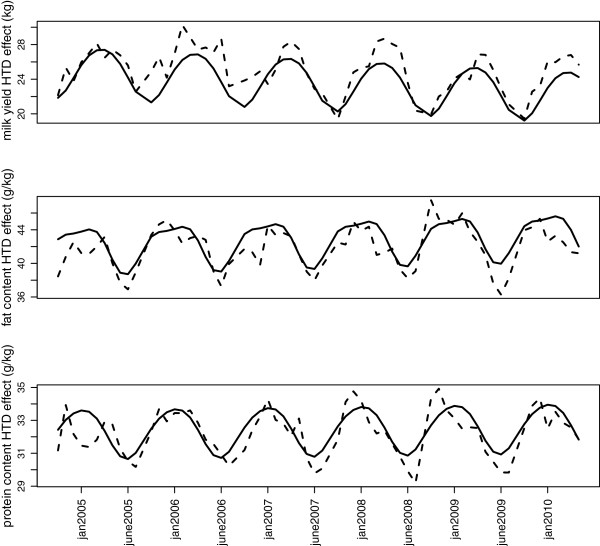
**Herd test profiles (figure extracted from **[1]**).** This figure shows an example of a herd described by its three HTD profiles (for milk yield, and protein and fat contents) before (dashed line) or after (solid line) smoothing.

Herd environments were described through HTD profiles for milk yield, fat and protein contents between 2005 and 2010. HTD profiles represent the evolution of HTD effects over time, as HTD effects are obtained from a test day model evaluation which aims at predicting the breeding value of animals at any day of the lactation period. The test day model uses each test day record available in national databases, in contrast to the 305-day lactation model which relies on the performance of an animal cumulated over 305 days. In order to improve the accuracy of daily breeding value estimation, other factors affecting the performance such as age and month of calving, length of dry period and gestation are estimated over the whole lactation through splines. Similarly, genetic and permanent environment effects throughout the lactation are predicted using continuous functions and the detailed description of the French test day model is given in [2]. The HTD effect is independent from all other effects and it estimates the effect of all features common to all cows of the herd on a particular test-day, i.e., essentially the effect of herd management (feeding, housing) of the test day. Therefore, the HTD effect can be interpreted as the herd management level of a herd on a given test-day. The HTD profile is a continuous function showing changes in HTD effects over time and can be interpreted as the changes in the herd management level over time. In previous studies, genetic evaluation using a test day model was carried out for milk yield and for fat and protein contents on French national data bases, separately for Holstein, Normande and Montbéliarde, the three major dairy breeds. This made it possible to describe herds by their three HTD profiles (milk yield, protein and fat contents) from 2005 to 2010 (see dashed curves in Figure 1).

HTD profiles, reflecting changes in HTD effects over time, can be decomposed into a systematic within year change that will be assumed to reveal practices related to the global herd management during the year as in [3], and a deviation from this global component due to specific characteristics (unusual weather conditions, feedstuffs availability, etc.) that cannot be related to regular management activities. Therefore, HTD profiles had to be corrected for these occasional features in order to be used as the definition of the environment in a G*E interaction study. For this purpose, HTD profiles were smoothed to focus on their repeated annual features using a model inspired by the model of Koivula *et al*[3] and described in [1]. Basically, the method consisted of describing HTD profiles by a continuous function involving a linear trend and three sine curves. Examples of HTD profiles before and after smoothing are shown in Figure 1. Note that in the rest of the study, only herds for which smoothing was obtained with a minimum coefficient of determination were retained (see [1] for details).

**Figure 2:**
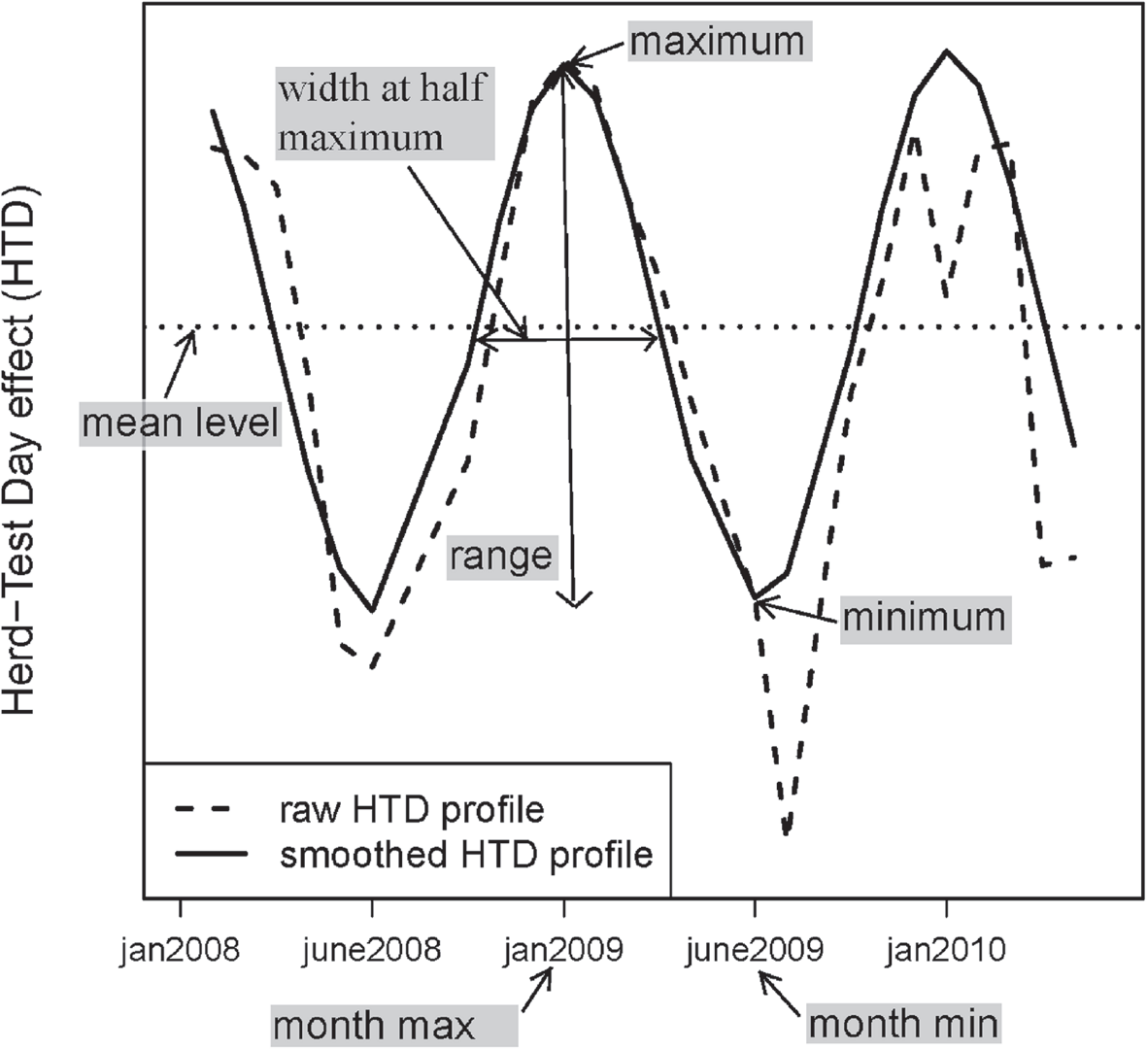
**Descriptors of herd test day profiles (figure extracted from **[1]**).** This figure shows the descriptors of smoothed HTD profiles used in the Multiple Factorial Analysis.

Each HTD profile was then summarized by seven descriptors, as shown in Figure 2, leading to 21 descriptors (7 descriptors times 3 traits) for each herd. These descriptors were reduced with Multiple Factor Analysis (MFA) to 10 Principal Components (PC) by retaining all PC that explained more than 4% of the total variance. MFA is similar to principal components analysis, which enables the joint use of categorical and quantitative data [4]. The MFA was performed on data from 12 061 Holstein, 2 591 Normande and 1 104 Montbéliarde herds. The descriptors were centered within breed in order to correct for breed effects.

The first principal component (PC1, explaining 15% of the total variance) was interpreted as a measure of the specialisation of the herd management; it discriminated herds with herd management favouring high milk production (low PC1 score) from the herds favouring high fat content (high PC1 score). The second PC (13%) was interpreted as a measure of the intensity of production related to herd management; it discriminated herds with high HTD effects for milk yield, and for fat and protein contents (high PC2 score) from herds with low HTD effects for milk yield, fat and protein contents (low PC2 score). Principal component 3 (8%) was interpreted as related to the seasonality of herd management. It differentiated herds for which the range of HTD profiles for the three traits was small (high PC3 score) from those with large ranges (low PC3 score), that is, PC3 discriminated herds in which herd management led to more or less similar milk yield and composition between seasons from herds in which herd management led to variable milk yield and composition across seasons. The next seven PC explained 46% of the total variance but their interpretation was less intuitive. In total, these 10 first PC explained 76% in HTD effects of the total variance and were mainly related to the mean level of HTD effects and the range of HTD profiles, rather than to periods during which minimum and maximum HTD effect were obtained.

Based on the MFA, herd environment was characterized by the 10 PC scores for 15 756 Holstein, Normande and Montbéliarde herds. These PC scores and their interpretations were the basis of the different herd environment definitions that were used to describe G*E interactions.

### Estimation of genotype by environment interactions

G*E interactions were estimated for the three breeds based on data from two different sets of herds within breed (“paragon” or “diversity”, see below) with two different models (multiple-trait and reaction norm models). For clarity, only analyses and results for the Normande and Holstein breeds, which are respectively a national dual purpose and an international dairy breed, are presented here. The following paragraphs describe the methodology used.

#### Herd selection for G*E estimation

Studies on the estimation of G*E interactions require the estimation of genetic parameters, which was carried out within breed (Holstein, Normande). A substantial but not excessively large data set is required for this purpose in order to obtain accurate estimates but limit computation time. Among the 12 061 Holstein and 2 591 Normande available herds, herds used in the G*E interaction study were selected by two strategies. Both were based on the PC scores of the herds. The first strategy consisted in selecting only typical herds for which characteristics were representative of a majority of the French herds, leading to what will be referred hereafter as the “paragon herd data set” (a paragon is regarded as a perfect example of a particular feature). The second strategy aimed at representing the complete diversity of French herds, leading to the “diversity herd data set”.

**Figure 3:**
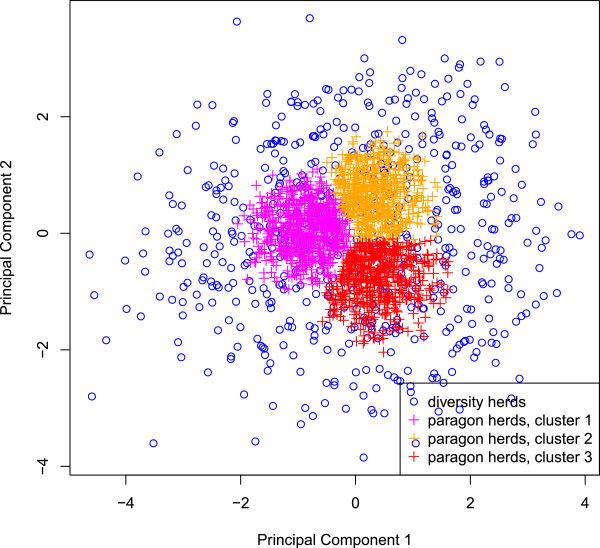
**Diversity and paragon herds for the Holstein breed on the first factorial map.** This figure shows how Holstein herds from the diversity and paragon datasets were distributed over the first factorial map. Herds in pink, orange and red are the “paragon” herds and depict the three herd clusters used in the multiple-trait model for the paragon dataset.

**Figure 4:**
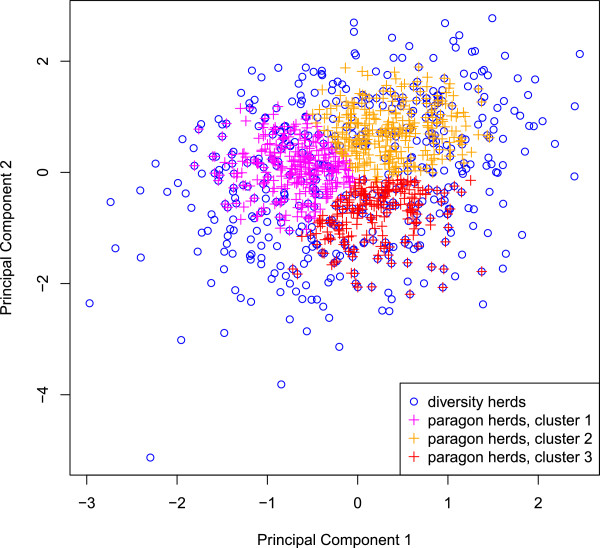
**Diversity and paragon herds for the Normande breed on the first factorial map.** This figure shows how Normande herds from the diversity and paragon datasets were distributed over the first factorial map. Herds in pink, orange and red are the “paragon” herds and depict the three herd clusters used in the multiple-trait model for the paragon dataset.

For the first strategy, three herd groups were created using a clustering method based on the first 10 factors of the MFA. Note that this was not a classification approach. See [1] for more methodological details. At this stage, each cluster included herds of the three breeds. However, since the G*E analysis was carried out within breed, herd clusters were then defined per breed. Practically, Normande and Holstein herds of cluster 1 in the joint breed dataset were respectively assigned to cluster 1 in the Normande and Holstein datasets, and the same for clusters 2 and 3. These herd clusters were also used as classes for the definition of fixed effects in the reaction norm model. The 400 Normande and 750 Holstein herds that were most representative herds of each cluster, i.e. those nearest to the center of each cluster, were selected as paragons and included in the “paragon dataset”. They are represented in pink, orange and red in Figures 3 and 4 for the Holstein and Normande breeds, respectively. This strategy was primarily used to obtain clearly distinct and well-defined clusters for the G*E interaction estimation by a multiple-trait model. Indeed, defining clusters of herds along a continuum and then choosing the paragons in each cluster forced some level of homogeneity within and heterogeneity between clusters. This increased the power to detect possible G*E interactions with the multiple-trait model. The “paragon dataset” was also used with the reaction norm models in order to compare both models on the same dataset.

For the second strategy, the first factorial map (PC 1 and 2 of the MFA) was divided into 25 equal-sized cells. Up to 40 or 60 herds (for the Holstein and Normande breeds, respectively) were selected at random for each cell. This led to two “diversity data sets” containing respectively 539 Holstein and 472 Normande herds (in blue in Figures 3 and 4, respectively).

#### Data selection for estimation of G*E interactions

The traits analysed were 305-day milk, fat and protein yield, as well as 305-day fat and protein contents. Data consisted of first lactations from cows which had a first calving between 2000 and 2011. Records were edited on several criteria: performances deviating by more than three phenotypic standard deviations from the breed average, herds with less than 100 first lactations from 2000 to 2011, records from daughters of bulls with less than three daughters that were located in just one herd were deleted, as well as records from cows which had an age at first calving lower than 23 months or greater than 40 months, or a lactation length lower than 180 days. These steps reduced the size of the datasets. Tables 1, 2 and 3 show the final number of herds and records used to estimate G*E interactions for the different models, breeds and herd data sets.

**Table 1:** Characteristics of the Holstein paragon dataset for the multiple-trait model

	**Herd cluster**	**C1**	**C2**	**C3**
	Number of herds	629	555	506
Data	Number of records	99,862	79,104	67,774
	Pedigree	412,732		
	PC 1 score:			
	herd management specialisation	-0.88 (0.4)	0.36 (0.4)	0.38 (0.5)
Environment	milk yield vs protein and fat contents			
descriptors	PC 2 score:	0.06 (0.4)	0.69 (0.4)	-0.79 (0.4)
	herd management intensity			
	PC 3 score:	0.15 (0.4)	0.05 (0.5)	-0.02 (0.5)
	herd management seasonality			
	Milk yield (kg)	10,163 (1,550)	9,818 (1,553)	9,152 (1,449)
	Fat yield (kg)	399 (60)	392 (62)	364 (59)
Phenotypes	Protein yield (kg)	325 (49)	316 (50)	291 (47)
	Fat content (%)	3.95 (0.43)	4.01 (0.42)	4.0 (0.42)
	Protein content (%)	3.20 (0.20)	3.23 (0.20)	3.19 (0.20)

This table presents the number of herds, of first lactation records, of animals included in the pedigree file and the mean and standard deviation (between brackets) of analysed traits and PC scores (only for the 3 first PC; herd clusters were created based on the first 10PC) for each herd cluster and breed.

**Table 2:** Characteristics of the Normande paragon dataset for the multiple-trait model

	**Herd cluster**	**C1**	**C2**	**C3**
	Number of herds	291	287	218
Data	Number of records	49,131	45,352	30,982
	Pedigree	197,039		
	PC 1 score:			
	herd management specialisation	-0.70 (0.4)	0.36 (0.4)	0.21 (0.4)
Environment	milk yield vs protein and fat contents			
descriptors	PC 2 score:	0.08 (0.5)	0.75 (0.4)	-0.75 (0.5)
	herd management intensity			
	PC 3 score:	-0.14 (0.5)	-0.23 (0.5)	-0.07 (0.6)
	herd management seasonality			
	Milk yield (kg)	7,649 (1,219)	7,438 (1,230)	6,935 (1,162)
	Fat yield (kg)	327 (54)	322 (55)	297 (52)
Phenotypes	Protein yield (kg)	266 (43)	259 (44)	239 (41)
	Fat content (%)	4.29 (0.37)	4.34 (0.36)	4.30 (0.36)
	Protein content (%)	3.48 (0.20)	3.49 (0.20)	3.15 (0.20)

This table presents the number of herds, of first lactation records, of animals included in the pedigree file and the mean and standard deviation (between brackets) of analysed traits and PC scores (only for the 3 first PC; herd clusters were created based on the first 10 PC) for each herd cluster and breed.

**Table 3:** Characteristics of the paragon and diversity datasets for the reaction norm model

	**Breed**	**Holstein**		**Normande**	
	**Dataset**	**Paragons**	**Diversity**	**Paragons**	**Diversity**
	Number of herds	1,690	539	796	472
Data	Number of records	246,740	75,173	125,465	70,105
	Pedigree	412,732	133,310	197,039	112,276
	PC1 score: herd management specialisation	-0.13 (0.7)	-0.12 (1.7)	-0.10 (0.6)	-0.07 (1.0)
Environment	milk yield vs protein and fat contents				
descriptors	PC2 score: herd management intensity	0.03 (0.8)	0.08 (1.5)	0.12 (0.7)	0.01 (1.2)
	PC3 score: herd management seasonality	0.07 (0.5)	0.10 (0.8)	-0.16 (0.5)	-0.12 (0.8)
	Milk yield (kg)	9,775 (1,577)	9,777 (1,863)	7,397 (1241)	7,294 (1,321)
	Fat yield (kg)	387 (62)	386 (73)	318 (55)	314 (59)
Phenotypes	Protein yield (kg)	313 (51)	313 (60)	257 (44)	253 (48)
	Fat content (%)	3.99 (0.43)	3.98 (0.45)	4.31 (0.36)	4.32 (0.37)
	Protein content (%)	3.21 (0.20)	3.20 (0.20)	3.48 (0.20)	3.46 (0.21)

This table displays the number of herds, of first lactation records, of animals used in the pedigree file and the mean and standard deviation (between brackets) of traits and environmental descriptors.

#### Models

Two models to estimate G*E interactions were tested: a multiple-trait model, in which the environment was considered to be specific to a group of herds, and a reaction norm model, in which the environment varied continuously as a function of PC scores of the herds. Both were animal models from the perspective of fitting breeding values. The pedigree files contained three generations (see Tables 1, 2 for the multiple-trait model and Table 3 for the reaction norm model). The only difference between the two models was the modelling of breeding values and additive genetic variance; fixed effects were the same for the two models. All analyses were carried out using the WOMBAT software [5], separately for each trait and breed. A more detailed description of the two models follows:

∙ Multiple-trait model

The multiple-trait across country evaluation (MACE) [6] can be seen as a G*E analysis for which the environment is country-specific. A given trait (e.g. milk production) recorded in different countries is modelled as distinct traits. Consequently, it is named a multiple-trait model, although a single phenotype is analysed. Using this model, genetic correlations can then be estimated between countries/environments. The model used in this paper mimics the model proposed in [7], in which the environment is defined by herd clusters. It was applied to the paragon datasets of each breed. Environments were defined based on the three herd clusters identified in the first strategy of herd selection (basis of the “paragons herd data set”). These are shown in pink, orange and red in Figures 3 and 4 for the Holstein and Normande breeds, respectively. Tables 1 and 2 summarize the average performances for these three herd clusters in the paragon dataset for the Hosltein and the Normande breed respectively.

The multiple-trait model was:

$$\begin{array}{ll} Y_{\text{ih}}\;=\;{\text{HerdYear}}_{i}+{\text{AgeYearCluster}}_{i}\\ \;\;\;+{\text{MonthYearCluster}}_{i}+a_{\mathrm{ic}}+e_{\text{ci}}\; & \; \end{array}$$

where: *Y*_*ih*_ = 305-day first lactation performance of animal *i* in herd *h*, HerdYear is the fixed effect of Herd-Year class, AgeYearCluster is the fixed effect of Age at calving-Year-herdCluster class, MonthYearCluster is a fixed effect of Month at calving-Year-herdCluster subclass, *a*_*ic*_ is the random genetic effect of cow *i* in the herd cluster *c*, and *e*_*ci *_is the random residual effect. Note that genetic and residual variances were different for each herd cluster. This model showed problems of convergence whatever the Restricted Maximum Likelihood (REML) estimation algorithm used (combinations of options Average Information (AI), Parameter Expanded (PX) and Expectation Maximisation (EM) of the WOMBAT software [5]). Some eigenvalues of the genetic variance/covariance matrix were close to zero, because all genetic correlations estimated between herd clusters were very close to one. Consequently, the following single-trait model was implemented instead:

$$\begin{array}{l} Y_{\text{ih}}\;=\;{\text{HerdYear}}_{i}+{\text{AgeYearCluster}}_{i}\\ \;\;\;+{\text{MonthYearCluster}}_{i}+a_{i}+e_{\text{cyi}}. \end{array}$$

The trait is assumed to be the same genetic trait in each cluster but the model allows for different genetic variances by cluster. The same fixed effects were estimated as in the previous model. Heterogeneous residual variance (*e*_*cyi*_) by the combination of herd cluster and birth year period was allowed for rather than only by herd cluster. Birth year periods were based on years 2000 to 2002, 2003 to 2005, 2006 to 2008 and 2009 to 2011. Hence, four residual variances were obtained for each herd cluster; one for each group of three birth years. The mean of these four variances was used as the overall residual variance of the herd cluster. Finally, heritabilities for each herd cluster were calculated as the ratio of the genetic variance to the sum of the genetic and residual variances for that herd cluster.

∙ Reaction norm model

In reaction norm models, the additive genetic effect is described as a continuous function of environmental parameters. In this study, PC scores of herds were used as environmental parameters. The reaction norm model was applied both to the “paragon” and “diversity” herd datasets. Table 3 summarizes average performances and environment descriptors (PC scores) for both sets of herds and breeds.

The model was :

$$\begin{array}{ll} Y_{\text{ih}}\;=\;{\text{HerdYear}}_{i}+{\text{AgeYearCluster}}_{i}\\ \;\;\;+{\text{MonthYearCluster}}_{i}+a_{i}+e\; & \; \end{array}$$

with $a_{i}=a_{0i}+\overset{p}{\underset{j=1}{\sum }}(a_{\mathrm{ji}}*PC_{\mathrm{jh}})$.

The three herd clusters defined in the first herd selection strategy were included as fixed effects subclasses, whereas the breeding value of a cow was estimated as a function of the PC scores of the cow’s own herd, rather than the PC scores of the herd cluster. This study focused on random genetic effects across environments for the estimation of G*E interactions and not on the fixed effects. Using exactly the same fixed effects allowed comparison of estimates of G*E interactions between the single-trait and reaction norm models. Heterogeneous residual variances by group of birth year period (2000 to 2002, 2003 to 2005, 2006 to 2008 and 2009 to 2011) were estimated. Animal breeding values *a*_*i*_ were modelled in two parts: one (*a*_0*i*_) expressing the animal’s average effect across environments and one ( $\overset{p}{\underset{j=1}{\sum }}(a_{\mathrm{ji}}*PC_{\mathrm{jh}})$) that depended on the PC scores of the animal’s herd, i.e., on the environment. *PC*_*jh*_ is the PC score of herd *h* on the *j*^*th*^ axis of the factor analysis. *a*_*ji*_ is the coefficient applied for animal *i* on the *j*^*th *^PC score of its herd. Note that linear reaction norms were assumed. Analyses were carried out taking into account the first one, two or three PC scores (*p *= 1, 2 or 3). Within breed and trait, these three models were compared using the Bayesian Information Criterion (BIC). Only results from the best model (the one with the smallest BIC value) will be presented.

The estimated covariance matrix (V) of the reaction norm model combined variances and covariances of the random genetics effects *a*_0_, *a*_1_,.., *a*_*p*_ of, for example, milk yield:

$$V=\left[\begin{array}{ccc} \sigma_{a_{0}(\text{milk})}^{2} & & \\ \sigma_{a_{0}(\text{milk}),a_{1}(\text{milk})} & \sigma_{a_{1}(\text{milk})}^{2} & \\ \vdots & \;\;\;\;\;\ddots & \\ \sigma_{a_{0}(\text{milk}),a_{p}(\text{milk})} & \sigma_{a_{1}(\text{milk}),a_{p}(\text{milk})}\;\;\ldots & \sigma_{a_{p}(\text{milk})}^{2} \end{array}\right].$$

Genetic variances and covariances for each herd cluster were obtained using the expression *MVM*^*′ *^with:

$$M=\left[\begin{array}{ccccc} 1 & \mathrm{PC}1{\mathrm{score}}_{1} & \mathrm{PC}2{\mathrm{score}}_{1} & \ldots & {\mathrm{PCpscore}}_{1}\\ \vdots & \vdots & \vdots & \vdots & \vdots \\ 1 & \mathrm{PC}1{\mathrm{score}}_{n} & \mathrm{PC}2{\mathrm{score}}_{n} & \ldots & {\mathrm{PCpscore}}_{n} \end{array}\right].$$

A row of matrix *M* represented one “state” of the environment gradient, i.e. specific values of the environment, described by *p* PC scores, with *p* depending on the model. The number of environment states analysed were arbitrarily chosen to be respectively 25 and 625 for models with one or two PC, respectively, to describe the environment. Four residual variances were estimated per breed and trait; one for each of four birth year periods. The mean of these four residual variances was used as the reference residual variance. Finally, heritabilities for each state of the environment were calculated as the ratio of the genetic variance over the genetic plus residual variance for that state of the environment. Genetic correlations between two environment states were calculated as the ratio of the genetic covariance between these two environments to the product of their genetic standard deviations.

## Results

### Multiple and single-trait analysis with the paragon dataset

#### Description of the three herd clusters/environments

With the multiple-trait model, environment was described by three herd clusters which represented three types of herd management. They were built based on the first 10 PC scores, summarizing the features of their three HTD profiles. Therefore, herd clusters were built based on production level only due to herd management rather than the global production level (that includes herd management but also genetic effect for example). Thus in the following, the production level must be interpreted as the level of milk yield and protein and fat contents due to herd management only. The interpretation of herd clusters was very similar for both breeds. Tables 1 and 2 show the number of herds in each cluster and means of PC scores in each herd cluster for each breed. Cluster 1 was made up of herds with management that resulted in an average production intensity (mean PC2 score close to zero) but that was more geared towards milk production than towards protein and fat contents (negative PC1 score). The management of the herds in this cluster was rather insensitive to season of production for the Holstein breed (mean PC3 score is positive) but not for the Normande breed (mean PC3 score is negative). Cluster 2 consisted of herds with a higher herd management intensity (high PC2 score) and rather specialised in protein and fat contents (positive PC1 score). Management of herds in this cluster was affected by the season of production for the Normande breed but not for the Holstein breed. Cluster 3 was composed of herds with a low herd management intensity (negative PC2 score).

#### Genetic parameters in the three environments

In the multiple-trait model, which had problems of convergence, the smallest genetic correlation was 0.98 for protein content in the Normande breed between cluster 1 and cluster 3. This indicated that there was no reranking of animals across herd clusters, i.e., environments. The single-trait model assumed that genetic correlations were one between environments but allowed for heterogeneous genetic and residual variances, leading to different heritabilities across herd clusters (Table 4).

**Table 4:** Residual variance, genetic variance and heritabilities for the three herd clusters with the single-trait model

		**Holstein**			**Normande**		
**Trait**		**C1**	**C2**	**C3**	**C1**	**C2**	**C3**
	gen var	835,690	802,710	590,920	519,830	473,220	420,700
		(17,204)	(18,570)	(15,387)	(15,473)	(15,302)	(15,638)
Milk yield	res var	952,294	970,338	887,510	636,713	643,032	586,439
	*h*^2^	0.47	0.45	0.40	0.45	0.42	0.42
	gen var	1232.5	1193.7	939.62	822	821	744
		(26)	(29)	(25)	(27)	(29)	(28)
Fat yield	res var	1507	1605	1443	1260	1284	1141
	*h*^2^	0.45	0.43	0.39	0.39	0.39	0.39
	gen var	608	580	416	513	472	428
		(15)	(16)	(13)	(17)	(17)	(17)
Protein yield	res var	967	1013	896	757	771	685
	*h*^2^	0.39	0.36	0.32	0.40	0.38	0.38
	gen var	15.5	15.4	15.0	8.4	8.4	7.8
		(0.18)	(0.20)	(0.21)	(0.16)	(0.17)	(0.19)
Fat content	res var	3.9	4.2	4.2	3.6	3.9	3.9
	*h*^2^	0.80	0.78	0.78	0.70	0.69	0.67
	gen var	2.6	2.5	2.5	2.5	2.5	2.4
		(0.038)	(0.041)	(0.042)	(0.051)	(0.052)	(0.061)
Protein content	res var	1.3	1.4	1.4	1.2	1.3	1.3
	*h*^2^	0.67	0.64	0.64	0.67	0.66	0.64

Variances (with standard errors between brackets) and heritabilities are presented for each trait by breed and herd cluster (C1, C2, C3).

Except for fat yield in the Normande breed and fat content in the Holstein breed, heritabilities decreased from cluster 1 to cluster 3, for all traits and breeds: heritabilities were greater for herds with a high herd management intensity that favoured milk production. The largest ranges of heritabilities were found in the Holstein breed (Table 4): with a decrease in heritability from herd cluster 1 to herd cluster 3 by 15% for milk yield, 15% for protein yield, 22% for fat yield. In most cases, these decreases in heritability were due to a greater decrease of genetic variance than of residual variance.

In conclusion, the multiple-trait model did not reveal evidence of significant reranking of animals between environments. However, although herd clusters in the paragon dataset did not reflect extreme herd managements, heritabilities were found to differ between environments.

### Reaction norm model

Reaction norm models were tested using one, two or three PC as environmental parameter(s) within breed, trait and dataset (paragon or diversity herd sets). According to the Bayesian Information Criterion, the best model used only PC1 as environmental parameter (i.e., herd management specialisation: milk yield versus protein and fat contents) for protein and fat contents, whereas the best models for milk, protein and fat yields used both PC1 and PC2 (herd management specialisation and herd management intensity) as environmental parameters. This was the case for all breeds and both datasets.

The first eigenvalue of the covariance matrix (V) that combined variances and covariances of the random genetics effects *a*_0_, *a*_1_,.., *a*_*p*_(where *p *= 1,2,3 is the number of PC included as environmental parameters) was very high whatever the breed, trait and dataset. This eigenvalue corresponded mainly to the random genetic effect *a*_0_, which represents the part of the breeding value that does not depend on the environment. This eigenvalue represented a minimum of 99% of the sum of eigenvalues of the covariance matrix (V). This was a further argument supporting the quasi absence of G*E interactions for production traits, in terms of reranking.

Estimates of residual variances are shown in Table 5 and genetic correlations and heritabilities in Tables 6 and 7. Note that for these results, only environment states corresponding to herds in the dataset were taken into account. Indeed, Figures 3 and 4 show that some areas defined by PC1 and PC2 included no herds (e.g. in the top left corner of the figures). On average, genetic correlations between environments were very high for all breeds, traits, and datasets (see Table 6), supporting again the absence of reranking of animals across environments. Genetic correlations were higher between environments defined for the paragon dataset than between environments defined for the diversity dataset. This was because herds included in the paragon dataset were chosen to reflect herd managements that were common in France. In contrast, the diversity dataset also included herds that represented extreme environments. In the diversity dataset, the average genetic correlations were lower for the Holstein than for the Normande breed. This is due to the fact that the Normande herds available in the study reflected herd managements less extreme than the Holstein herds: the range of their PC scores was narrower than the one for Holstein herds (see Figures 3 and 4). The lowest genetic correlations were obtained with the diversity dataset for milk, fat and protein yields: between 0.60 and 0.68 for the Holstein breed and between 0.86 and 0.92 for the Normande breed. These correlations were obtained between extreme environments.

**Table 5:** Residual variances with the reaction norm model

	**Holstein**		**Normande**	
**Trait**	**Paragon**	**Diversity**	**Paragon**	**Diversity**
Milk yield	931,367	945,995	623,825	620,922
Fat yield	1,506	1,531	1,233	1,207
Protein yield	948	940	740	723
Fat content	4.12	4.63	3.74	3.75
Protein content	1.33	1.35	1.27	1.29

**Table 6:** Genetic correlations between environments with the reaction norm model

		**Holstein**						**Normande**					
		**Paragon**			**Diversity**			**Paragon**			**Diversity**		
**Trait**	**Model**	**Min**	**Mean**	**Max**	**Min**	**Mean**	**Max**	**Min**	**Mean**	**Max**	**Min**	**Mean**	**Max**
Milk yield	*RN*_2_	0.96	0.99	1	0.68	0.97	1	0.99	0.99	1	0.92	0.99	1
Fat yield	*RN*_2_	0.97	0.99	1	0.60	0.96	1	0.99	0.99	1	0.89	0.99	1
Protein yield	*RN*_2_	0.93	0.99	1	0.64	0.96	1	0.99	0.99	1	0.86	0.99	1
Fat content	*RN*_1_	0.99	0.99	1	0.94	0.99	1	0.98	0.99	1	0.99	0.99	1
Protein content	*RN*_1_	0.99	0.99	1	0.99	0.99	1	0.99	0.99	1	0.99	0.99	1

Minimum, mean and maximum estimates of genetic correlations between environments are given for each trait by breed and dataset.

**Table 7:** Heritabilities across environments with the reaction norm model

		**Holstein**						**Normande**					
	**Dataset**	**Paragon**			**Diversity**			**Paragon**			**Diversity**		
**Trait**	**Model**	**Min**	**Mean**	**Max**	**Min**	**Mean**	**Max**	**Min**	**Mean**	**Max**	**Min**	**Mean**	**Max**
Milk yield	*RN*_2_	0.26	0.43	0.55	0.12	0.42	0.66	0.28	0.43	0.52	0.17	0.40	0.53
Fat yield	*RN*_2_	0.28	0.42	0.52	0.15	0.40	0.63	0.27	0.39	0.47	0.14	0.37	0.50
Protein yield	*RN*_2_	0.80	0.35	0.49	0.07	0.36	0.64	0.24	0.39	0.48	0.12	0.36	0.52
Fat content	*RN*_1_	0.79	0.79	0.79	0.76	0.76	0.77	0.67	0.69	0.70	0.68	0.69	0.71
Protein content	*RN*_1_	0.65	0.66	0.67	0.63	0.64	0.66	0.65	0.66	0.67	0.64	0.65	0.66

Minimum, mean and maximum of heritabilities across environments are given for each production trait by breed and dataset.

Although no reranking was shown, heterogeneity of heritabilities was again found for milk, fat and protein yields for both breeds (Table 7), demonstrating a scale effect. Since the residual variance was the same across environments within breed, trait and dataset, this was due to a heterogeneity of genetic variances across environments. In contrast, heritabilities for protein and fat contents were more homogeneous across environments. Similar to what was observed for genetic correlations, the range of heritabilities was higher in the diversity dataset than in the paragon dataset and was even higher for the Holstein breed than for the Normande breed.

**Figure 5:**
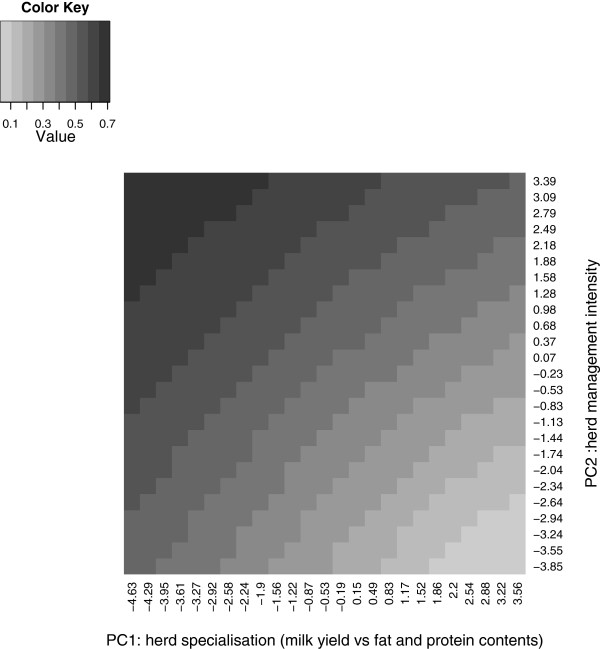
**Heritabilities for milk yield in the Holstein breed based on the reaction norm model.** This figure shows heritabilities of milk yield calculated from the reaction norm model applied on the Holstein diversity dataset.

**Figure 6:**
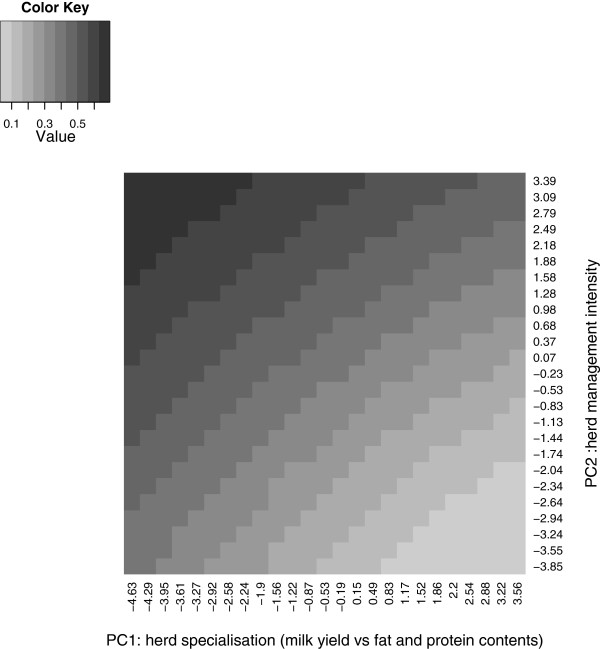
**Heritabilities for protein yield in the Holstein breed based on reaction norm model.** This figure shows heritabilities of protein yield estimated from the reaction norm model applied to the Holstein diversity dataset.

For the yield traits, environment was described simultaneously by two environmental parameters (PC1 and PC2 scores). The shape of heritabilities across environments was the same for all breeds and datasets for milk, protein and fat yields. Figures 5 and 6 show estimates of heritability for milk and fat yield, respectively, for the Holstein breed as functions of the PC1 and PC2 herd scores using the “diversity” dataset. Heritabilities increased with increasing PC1 and PC2 herd scores. This gradient was more important for herd management intensity (PC2) than for herd management specialisation (milk yield versus fat and protein contents, PC1).

For clarity, only analyses and results for the Normande and Holstein breeds were presented. However, estimates of G*E interactions in the Montbéliarde breed led to exactly the same conclusions with multiple-trait, single-trait and reaction norm models (results not shown).

## Discussion

The aim of this study was to estimate G*E interactions for production traits in the Holstein and Normande breeds in France. Genetic correlations between environments were very close to unity, except between very extreme environments for all breeds, models, and datasets, demonstrating that reranking of animals for production traits across environments does not exist in France. Such a result was previously reported in studies in France that used herd production level as definition of herd environment [8] as well as in other studies [9-11] that used different definitions of the environment. Yet, other studies did report genetic correlations less than one between environments, i.e., with reranking of animals. These studies dealt with data from different countries [12-15], that is, for ranges of environments that were greater than in this national study.

Variable genetic variances across environments for production traits were found in this study and have been reported before [9,16]. Genetic variances increased with the capacity of the herd management to promote milk production. These results are in agreement with [8,9,17], in which genetic variances increased with increasing production level.

In a G*E interaction study, the definition of the herd environment is crucial. Definitions used in the literature are extremely diverse; they depend on the scale of the study (experimental farm versus national or international studies) and on the traits analysed. In the case of production traits, definition of the herd environment can be based on specific features of the feeding system, such as the level of concentrate in the diet [18,19], grazing severity and silage quality [20], features of the reproduction system, such as the calving system (seasonal or uniform) [11], features of the herd structure, such as herd size [11], features of the climate such, as temperature humidity index [16], rainfall [7], or features of genetic background (percent of Holstein genes) [7]. Many studies have described the environment based on observed performances of the animals, such as herd milk production level [8,21], fat and protein yields [22], peak milk yield, or persistency [23]. In these cases, environmental and genetic factors are combined. In this study, herd environment was described based on HTD profiles. This definition focuses on the part of the production due to herd management only. This improves the study of G*E interactions because environmental and genetic factors are no longer confounded in the definition of the environment. Moreover, HTD profiles are available from national databases and do not require extra recording, in contrast to many herd management descriptors. Using HTD profiles allows analysis of large datasets. Finally, HTD profiles summarize all impacts of environment on production and offer a general overview of the environment, whereas some other herd management descriptors reduce environment to a limited number of features (temperature, average performances, herd size).

Summarizing HTD profiles descriptors by first PC scores allowed correlations between the 21 descriptors to be taken into account and a focus on the main causes of variability among HTD profiles. However, by limiting the analysis to the first PC scores, part of the diversity of HTD profiles that reflect differences in herd management (i.e., environment diversity) was not accounted for, regardless of the model used (multiple-trait, single-trait or reaction norm model). In fact, the three herd clusters used to describe the environment in the multiple-trait model were built based on the first 10 PC scores only, which explained about 76% of the total variance in HTD profiles. Moreover, we selected paragon herds for each cluster, which reduced the within-environment diversity. For the reaction norm models, the environment was described only through one or two PC scores. This may seem reductive but reaction norm models based on three PC scores gave poorer goodness of fit in terms of the BIC.

The models that were used to estimate G*E interactions were animal models with pedigree information over three generations, in contrast with other studies that used simpler models such as sire models [13,16] or sire-maternal grand sire models [17]. Two types of models were tested: multiple-trait and reaction norm models. A drawback of multiple-trait models is that they require classification of environments, which cannot represent the full diversity of environments. Moreover, in this study, the multiple-trait model was applied to the “paragon” dataset, in which extreme environments were not represented, which led to a reduction in environmental variance. Despite this reduced diversity of environments, a clear heterogeneity of heritabilities among the three herd clusters was identified. In contrast, reaction norms model an “infinite” number of environments, which more precisely depicts the existing continuum of the environment. Generally, the parameter that describes the environment in reaction norm models is a single measure such as age at calving, herd size [24], or herd-year averages of protein yield [25]. This environment parameter can also be a synthetic variable that summarizes information of several environmental variables. In [17], 65 environmental variables were reduced into four PC by a factor analysis and were used separately. Hence, one major improvement in the current study was that several PC were used simultaneously to describe the environment. The number of parameters to estimate in the model was limited by using linear reaction norms rather than more sophisticated functions such as polynomials. The next step will be to study the possibility to simultaneously account for a larger number of PC to describe the environment. In particular, a reduced rank genetic matrix could be used to summarize the effect of several PC on the genetic effect. Reaction norm models applied to the diversity dataset allowed the investigation of extreme environments (for one or two PC). Here, an average residual variance which did not depend on the environment was used to estimate heritabilities with the reaction norm model. Consequently, differences of heritabilities across environments were only due to differences in genetic variances, which may have exacerbated differences in heritabilities between environments. These differences of heritabilities across environments might be exacerbated by the use of linear reaction norms and of an average residual variance. Thus, the reaction norm model could be improved by allowing different residual variances across environments.

No reranking of animals was shown for production traits. These traits have been selected for a long time, and thus, animals may be well adapted to all herd managements that currently exist in France. Nevertheless, within the context of the development of a sustainable agriculture, new ecological constraints appear such as controlling the use of phytosanitary products or protecting some agricultural areas. Also, new economical constraints due to reorganization of agricultural areas, with a decrease in the number of farmers or the end of quotas and liberalisation of milk production could raise new types of herd managements. Depending on how breeders react to these constraints, the range of environments could get larger. Thus, G*E interaction studies will have to be updated in order to assess whether animals remain well adapted to all herd environments.

Greater G*E interactions might exist for more recently selected traits. For these traits, the processes may not have already removed the animals’ capacity to be specifically adapted to a particular environment. Thus, on a follow-up study, we will investigate G*E interactions on functional traits.

## Conclusions

Presence of G*E interactions was evaluated for production traits (milk, protein and fat yields, protein and fat contents) using multiple-trait (which was eventually converted to a single-trait model) and reaction norm animal models for the Holstein, Normande and Montbéliarde breeds, and using herd environment descriptors derived from HTD profiles. No reranking of animals between environments was found for any breed or model. Therefore, it can be concluded that existing breeding schemes are efficient regardless of the environment in which animals are raised and produce. However, a heterogeneity of heritabilities across environments was apparent. In most cases: the more intensive the herd management for milk yield, the larger the heritability. Ignoring this heterogeneity makes reliabilities of estimated breeding values inaccurate. Moreover, the heritability and the genetic variance gradients across environments could entail a higher genetic response in the most intensive herd managements.

## Competing interests

The authors declare that they have no competing interests.

## Authors’ contributions

BH and VD jointly conceived the design of the study. BH, HL and VD discussed the results. BH wrote and checked programs. BH wrote the draft of the manuscript, VD and HL made suggestions and corrections. All authors read and approved the final manuscript.
